# Explaining the impact of mHealth on maternal and child health care in low- and middle-income countries: a realist synthesis

**DOI:** 10.1186/s12884-021-03684-x

**Published:** 2021-03-09

**Authors:** Eveline M. Kabongo, Ferdinand C. Mukumbang, Peter Delobelle, Edward Nicol

**Affiliations:** 1 Division of Health Systems and Public Health, Stellenbosch University, Cape Town, South Africa; 2 Burden of Disease Research Unit, South African Medical Research Council, Cape Town, South Africa; 3 School of Public Health, University of the Western Cape, Cape Town, South Africa; 4 Department of Public Health, Vrije Universiteit Brussel, Brussels, Belgium; 5 Chronic Disease Initiative for Africa, University of Cape Town, Cape Town, South Africa

**Keywords:** mHealth, Mobile phone, Maternal health, Child health, Low- and middle-income countries, Realist synthesis

## Abstract

**Background:**

Despite the growing global application of mobile health (mHealth) technology in maternal and child health, contextual factors, and mechanisms by which interventional outcomes are generated, have not been subjected to a systematic examination. In this study, we sought to uncover context, mechanisms, and outcome elements of various mHealth interventions based on implementation and evaluation studies to formulate theories or models explicating how mHealth interventions work (or not) both for health care providers and for pregnant women and mothers.

**Method:**

We undertook a realist synthesis. An electronic search of five online databases (PubMed/Medline, Google Scholar, Scopus, Academic Search Premier and Health Systems Evidence) was performed. Using appropriate Boolean phrases terms and selection procedures, 32 articles were identified. A theory-driven approach, narrative synthesis, was applied to synthesize the data. Thematic content analysis was used to delineate elements of the intervention, including its context, actors, mechanisms, and outcomes. Abduction and retroduction were applied using a realist evaluation heuristic tool to formulate generative theories.

**Results:**

We formulated two configurational models illustrating how and why mHealth impacts implementation and uptake of maternal and child health care. Implementation-related mechanisms include buy-in from health care providers, perceived support of health care providers’ motivation and perceived ease of use and usefulness. These mechanisms are influenced by adaptive health system conditions including organization, resource availability, policy implementation dynamics, experience with technology, network infrastructure and connectivity. For pregnant women and mothers, mechanisms that trigger mHealth use and consequently uptake of maternal and child health care include perceived satisfaction, motivation and positive psychological support. Information overload was identified as a potential negative mechanism impacting the uptake of maternal and child health care. These mechanisms are influenced by health system conditions, socio-cultural characteristics, socio-economic and demographics characteristics, network infrastructure and connectivity and awareness.

**Conclusion:**

Models developed in this study provide a detailed understanding of implementation and uptake of mHealth interventions and how and why they impact maternal and child health care in low- and middle-income countries. These models provide a foundation for the ‘white box’ of theory-driven evaluation of mHealth interventions and can improve rollout and implementation where required.

**Supplementary Information:**

The online version contains supplementary material available at 10.1186/s12884-021-03684-x.

## Background

The potential for mobile health (mHealth) to enhance healthcare utilization, promote affordability and support accountability of health care in low-and middle-income countries (LMICs) is supported by the near-universal availability of mobile phones with increasing coverage in many LMICs [1, 2]. mHealth is described as an element of electronic health used for the provision of healthcare services using information and communication technology [3]. mHealth offers a personalized and interactive tool aimed at promoting healthcare access and awareness [4, 5]. mHealth also has the potential to strengthen public sector care for optimal management of chronic conditions and improvement of maternal and child health (MCH) care [6–8]. In addition to promoting health education among patients and reducing waiting times and costs of healthcare, mHealth enhances patient support, providing a system for emergency response and monitoring [7].

Potential challenges faced by mHealth interventions have been highlighted in previous studies [9, 10]. One-way mobile phone messaging is the most common type of mHealth communication used in LMICs [9]. A limitation of this approach, however, is that patients only receive messages and cannot interact with health care providers (HCPs) in real-time. Factors influencing mHealth interventions at individual level include users’ intentions, skills, attitudes, perceived norms, self-efficacy, literacy levels and proficiency in the use of mobile devices such as smartphones [9, 10]. Systems-related factors affecting the use of mHealth interventions include unsuitable implementation context, poor internet infrastructure, unreliable power supplies and frequent power outages.

Systematic reviews support the value of mHealth applications as an effective tool to improve MCH related outcomes, suggesting that it can be a key step towards achieving the Sustainable Development Goals (SDGs), in particular SDG 3 [8, 11] mHealth has shown to facilitate utilization of MCH services, increase clinic attendance and promote health-seeking behavior [12]. mHealth also supports regular immunization and exclusive breastfeeding by targeting behavioral change [8, 13].

Hackett et al., established that mHealth is significantly associated with MCH outcomes [14, 15]. While outcomes-based evaluation of mHealth interventions can offer insight into their performance, replicating findings across socio-demographic and geographical boundaries becomes challenging because mHealth interventions take on different forms. Having a functional understanding of how and why these interventions work (or not) can offer better implementation prospects. We sought to respond to this need by exploring and conceptualizing contextual elements and mechanisms that interact to explain observed effects of mHealth interventions on the uptake of MCH care in LMICs. We aimed to formulate models explicating how mHealth interventions work for HCPs and pregnant women and mothers by uncovering context, mechanisms and outcome elements in implementation and evaluation studies of mHealth interventions in MCH care in LMICs [3].

## Material and methods

Our study was informed by the critical realist understanding of generative causality as conceptualized by Pawson and Tilley [16]. To address the question: ‘What works, for whom, why, in what situation, and how?’ with regards to intervention, programs and policies. They proposed the formula Context (C) + Mechanism (M) (resource + reasoning) = Outcome (O) to express the relationship between context, mechanism and outcomes to explicate how interventions lead to behavior change or sustenance. According to this formula, O is a product of M in a specific C [16], and theories or models can be formulated, tested, confirmed and modified using a context-mechanism-outcome configuration (CMOc) [17]. Some implementation scientists have suggested modifications of the CMOc heuristic to improve its explanatory power [18, 19]. Marchal et al. [20] and Mukumbang et al. [21] proposed adding “intervention” (I) modalities and relevant “actors” (A) to the CMO configuration based on the fact that interventions (I) can only work when adopted by actors (A). Based on this modification, generative understanding postulates that “outcome (O) is produced by mechanism (M) activated in context (C) through actors (A) when interventions (I) are executed” [3, 22]. Models developed in this study were achieved by formulating Intervention-Context-Actors-Mechanism-Outcome (ICAMO) configurations (Table 1).

**Table 1:** Definition of the concepts in the ICAMO heuristic

Concepts	Definition/descriptions
Intervention (I)	Refers to the characteristics of various mHealth interventions such as type of technology, co-interventions, and modalities. In this case, mHealth modality was defined as use of mobile phones and tablets, making use of text, audio, images, short messaging services (SMS), voice SMS, applications accessible via general packet radio service.
Context (C)	Describes conditions required for programme mechanisms to activate or not. Context can be viewed as circumstances that facilitate or constrain mechanisms, including pre-existing individual, organisational, social and cultural conditions, that are external to the interventions [23]. In this case, context is categorised as a) Environmental, which comprises the broad external environment in which interventions are situated, including political, economic, social, technological, legal, and infrastructural environments [2]; and b) Organisational/health systems, which include resources, policies and structures directly related to the unique health facility settings in which mHealth technology is introduced [2].
Actors (A)	Includes individuals, groups, and institutions that play a role in the implementation and uptake of interventions [24]. In this study, actors include pregnant women, mothers and HCPs, including community health workers.
Mechanism (M)	A mechanism refers to causal forces, powers, processes or interactions that generate behavioural change. In realist evaluation terms, mechanisms include choices, perceptions, reasoning and decisions that people make as a result of the resources provided by programmes.
Outcomes (O)	Defined as products of mechanisms activated within specific contexts. Outcomes are anticipated and unanticipated (emergent) consequences of interventions [17].

**Fig. 1:**
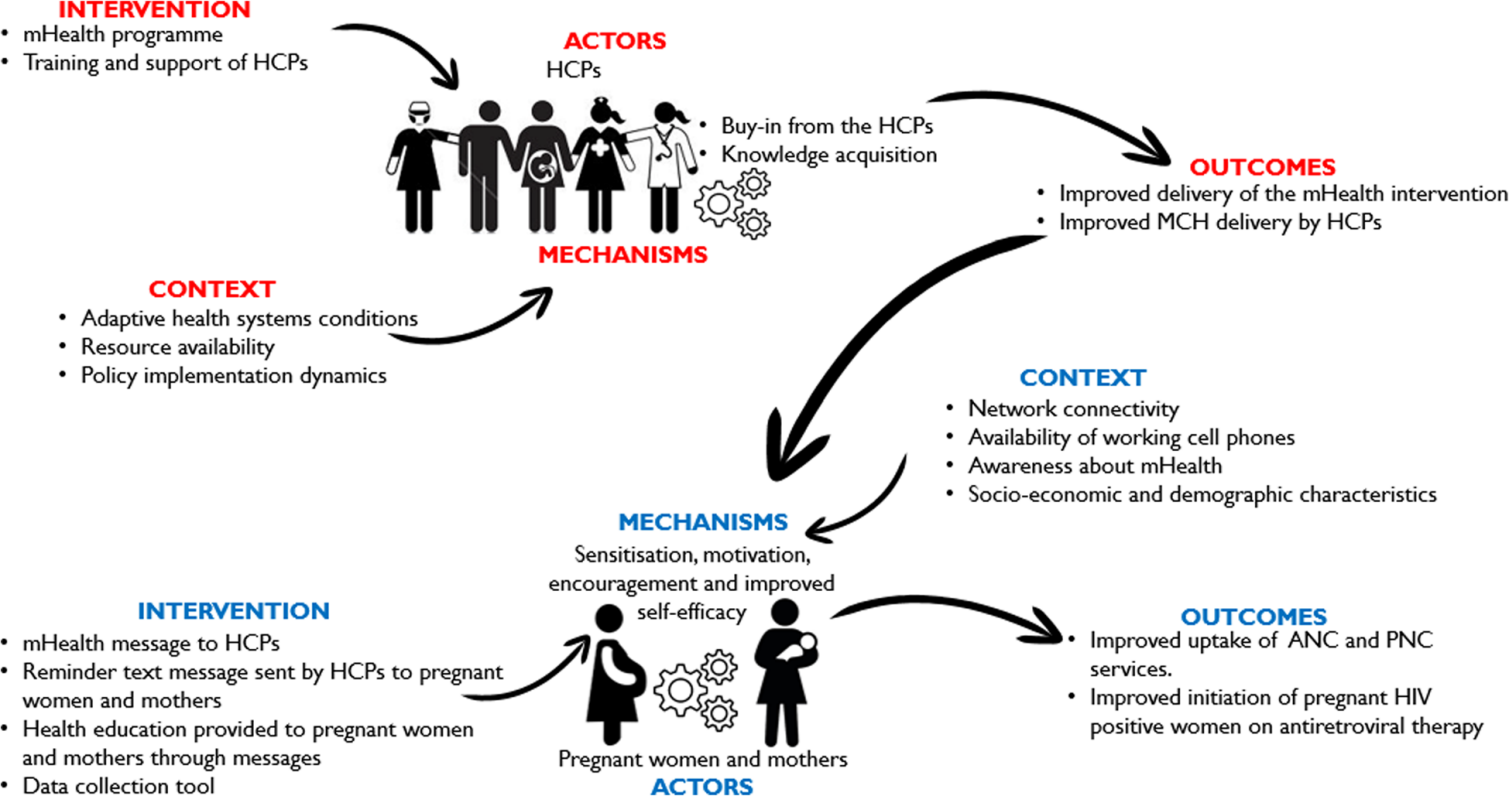
Tentative program theory of mHealth programs

Figure 1 shows a tentative conceptual model developed a priori based on existing literature on mHealth and MCH. This was achieved through abductive thinking – the inventive thinking required to imagine the existence of such mechanisms to ‘suggest’ the most likely possible explanation. The model suggests that when HCPs (A) are educated on mHealth interventions and trained on how to use programme resources (I), their perceived support will motivate (M), encourage (M) and improve their self-efficacy (M), in turn improving MCH care (O). Regarding to program users, the framework proposes that health educational and reminder messages of MCH (I) will sensitize, motivate (M) and encourage (M) pregnant women and mothers (A) to routinely use MCH care, such as emergency obstetric care, facility births (O) and early initiation of antiretroviral therapy for HIV positive women (O). We adopted a realist synthesis approach based on Pawson’s practical steps for conducting realist reviews [25], which include five stages, now addressed subsequently.

### Stage 1: clarifying the scope of the review

The purpose of the review is to determine how, why, for whom and under which conditions mHealth supports MCH care in LMICs. Initial scanning of the literature and expertise of the research team helped to define the research questions: 1) What mechanisms and contextual factors lead to the implementation and uptake of MCH care? 2) How do those mechanisms and contextual factors interact to explain implementation and uptake of MCH care?

### Stage 2: searching for relevant evidence

Five electronic databases (PubMed/Medline, Google Scholar, Scopus, Academic Search Premier and Health Systems Evidence) were searched for articles published between June 2008 and December 2018 using the following Boolean combinations: [“mHealth” AND “maternal health”], [“mobile phone” AND “maternal health” AND “child health”], [“mHealth AND “maternal health services”], [mHealth PRE/15 maternal] and [mHealth PRE/15 maternal AND child AND health]. A total of 813 records were identified.

Inclusion criteria were: peer-reviewed articles, published in English, published between January 2008 and June 2018 studies conducted in LMICs; studies targeting pregnant women, mothers with new babies and HCPs, including community health workers (CHWs). We considered cross-sectional, cohort, case-control and experimental studies, as well as randomized control trials.

Non-full text papers, technical reports, special reports, brief communications, presentation of scenarios or training workshops, editorial comments, non mHealth applications, telemedicine and other eHealth programme applications were excluded. Studies published before January 2008 were excluded as mHealth interventions were not common before that time.

### Stage 3: study selection and appraising quality of evidence

**Fig. 2:**
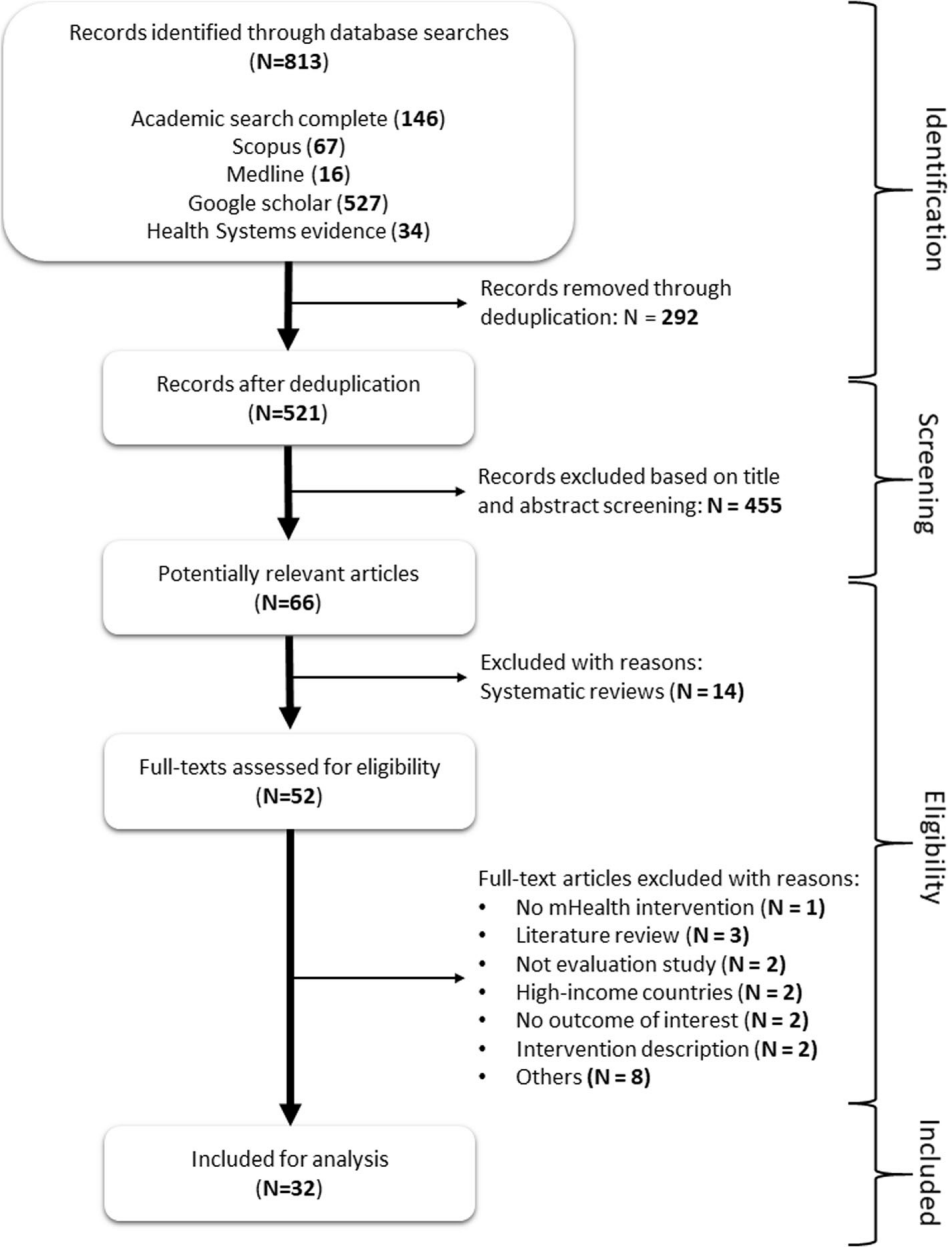
*PRISMA diagram illustrating the study selection process*

From 813 records in the database searches, 747 duplicates and non-relevant titles and abstracts were removed. Of the remaining 66 articles, 14 systematic reviews were also excluded. Fifty-two (*n* = 52) full-text articles were screened for potential inclusion and twenty (*n* = 20) were excluded for various reasons, yielding 32 articles (Fig. 2).

Quality assessment was performed for each article using a research evidence appraisal tool [26] (Supplementary File 1). Eight of the 32 articles were of high quality and 24 were classified as having good or moderate quality. Results from these studies could thus provide relevant and credible information towards challenging or enhancing the initial theory.

### Stage 4: extracting the data

Data were extracted and organized through a process of note-taking, annotation and conceptualization using the following headings: name of authors, year of publication and study setting or country; summary of the study aim; intervention, context, actors, mechanisms, and outcomes (Supplementary File 2).

### Stage 5: synthesizing evidence and concluding a process of reasoning

The narrative synthesis (NS) approach informed the process of collating, summarizing and reporting the results [27]. NS proposes a theory-driven approach to data synthesis and is compatible with the philosophical assumptions guiding theory formulation in realist evaluation [28]. NS relies on application of various methods of inference making through the use of words and text [27]. To this end, NS is applied in reviews addressing several questions with research evidence in the context of studies that strive to inform policy and practice [27]. Four interrelated steps are involved in NS: (i) Theory development of how interventions work: (ii) development of a preliminary synthesis of results; (iii) exploring associations in the data; and, (iv) assessment of the rigor of the synthesis.

#### Step 1. Theory development of how interventions work

According to Arial et al. [29], a thinking framework herein referred to as initial programme theory, is required as a first step to continuously test and revise our understanding of how mHealth interventions could improve MCH outcomes [30]. This initial program theory – an assumption of how the program should work – guides the process of operationalizing mechanisms into theories or models at the end of synthesis (see Fig. 1).

#### Step 2. Development of preliminary synthesis of results

We applied a deductive thematic analysis to extract data [31, 32] based on the concepts outlined in the ICAMO heuristic tool [33] and used an inductive approach to code constructs within each concept (Supplementary File 2). We identified relevant aspects of interventions (I), context factors (C), mechanisms (M) and outcomes (O) related to delivery of mHealth programs for CHWs and HCPs, and pregnant women and mothers.

#### Step 3. Exploring associations in the data

The realist evaluation approach [17, 34] informed the process of constructing the explanatory model. Three different methods were employed to establish associations of the extracted ICAMO themes: retroductive inferencing, counterfactual thinking and configuration mapping. We applied retroductive inferencing to explore the relationship between the themes of the ICAMO heuristic tool. Retroductive inferencing is a mechanism-focused analytical approach used to reconstruct the basic conditions of phenomena, based on available data (abductive reasoning). Counterfactual thinking was applied to argue towards transfactual conditions – the existence of powers, potentials and liabilities which cause the outcomes [31]. We then mapped possible explanations based on the data through the process of configurational mapping – a process of organizing and representing knowledge by linking and specifying relationships between variables.

#### Step 4. Assessment of the rigor of the synthesis

To assess robustness, we applied transparency, accuracy, purposively, utility, propriety, accessibility, and specificity (TAPUPAS) criteria (Table 2), an appraisal tool developed by Pawson et al. [35] to appraise the articles for relevance and to add more strength to the appraisal tool to assess the quality of the study (Supplementary File 1). Two study authors (EMK and FCM) applied judgmental rationality – the ability to evaluate different positions as being better or worse – to map ICAMO elements using Vensim® software [36] . This was achieved through discursive and iterative consultation among the researchers until consensus was reached.

**Table 2:** TAPUPAS criteria

Criteria	Guiding question
Transparency	Is it to scrutiny?
Accuracy	Is it well-grounded?
Purposive	Is it fit for purpose?
Utility	Is it fit for use?
Propriety	Is it legal and ethical?
Accessibility	Is it intelligible?
Specificity	Does it meet source-specific standards?

## Results

Thirty-two (32) studies from different geographic areas were identified: sub-Sahara Africa (21), Asia Pacific (10) and Latin America (1) (Supplementary File 2). Following the initial program theory (Fig. 1), findings are presented for HCPs and pregnant women and mothers. Out of the 32 studies, 20 contributed to the development of a model for HCPs, while 29 contributed to the model for pregnant women and mothers. We used Supplementary File 2 to extract data from the selected articles and the thematic analysis of the data are presented in Tables 3 and 4.

**Table 3:** Thematic representation of ICAMO element of HCPs

Variables	Themes
Intervention	**Communication platform** ▪ Information and education [5, 37–44]
	**Data management platform**
	▪ Registration, tracking, data collection and security [14, 45–49]
	**Decision support and guideline** [50–53]
Context	**Health system organisation**
	▪ HCPs training, supervision, resource availability, support and mobilisation [5, 14, 37, 40, 43, 45, 47, 48, 51–53]
	▪ Mobile phone availability and distribution to HCPs [38]
	▪ Availability of HCPs [39, 49]
	▪ HCPs and CHWs collaboration [39]
	**Socio-demographic characteristic**
	▪ Individual, pre-existing HCPs level of education, [41, 54]
	▪ Language spoken by HCPs [50, 53]
	**Experience with technology**
	▪ Technology adoption [37, 41]
	**Network infrastructure and connectivity**
	▪ Availability of network and connectivity [44, 50]
Actors	▪ HCPs
Mechanism	**Perceived support of HCPs**
	▪ Quality of training, resources, and administrative support impact on respondents’ task [40, 47]
	▪ Improved HCP-community relationship [5, 51]
	**Motivation**
	▪ Encouragement to be more active in performing tasks [38, 41, 42, 50]
	▪ Knowledge acquisition and skills gained improved self-efficacy and confidence [5, 14, 38, 39, 43, 45, 48, 49, 52]
	**Perceived ease of use and usefulness of mHealth**
	▪ [46, 47]
Outcomes	**Improved HCPs performance of care**
	▪ Improved accuracy in diagnosis, referral and recommendations [48, 52, 53]
	▪ More procreative [38, 39, 42], improved skills and help to overcome barriers [47]
	▪ Increase in rate of care attendance [40, 54]
	▪ Data security [51]
	**Improved quality of health care**
	▪ Improved MCH care [43] [50] ▪ Improved relation between HCPs and community members [5, 41]

**Table 4:** Thematic representation of the ICAMO element of pregnant women and mothers

Variables	Themes
Intervention	**Reminder messages system** [37, 50, 52, 55–61]
	**Communication platform**
	▪ Health information and education [5, 14, 15, 38, 39, 42, 43, 45, 46, 49, 50, 59, 62–65]
	**Consultation platform with HCPs** [39, 48, 51]
Context	**Health system aspects and political clout**
	▪ Government support [61]
	▪ Awareness of intervention [40, 63]
	▪ Availability of HCPs [38, 39]
	▪ Training, support, and supervision of HCPs [5, 45, 48]
	▪ Health system responsiveness [40, 43, 49, 60]
	**Socio-cultural characteristics**
	▪ Socio-cultural practices, social structures, and norms [14, 61, 64]
	▪ Community buy-in [51]
	**Socio-economic and demographic characterises**
	▪ Pre-existing individual (education, health literacy) characteristics [15, 37, 42, 46, 50, 52, 56, 57, 59, 62, 64–66]
	▪ Income [14, 55]
	▪ Access to a cell phone [46, 50, 55, 56, 58, 62, 65, 66]
	**Technical aspects of Mobile phone**
	▪ Access to a working phone [46, 50, 55, 56, 58, 62, 65, 66]
	▪ Network availability and connectivity [15, 39, 56, 63]
	▪ Preferences of language [58]
	▪ Lack of trust in technology and face-to-face preference [61]
Actors	**Pregnant women and mothers**
Mechanism	**Perceived satisfaction**
	▪ Satisfaction with care [48, 56, 60, 63]
	▪ Perceived privacy and confidentiality [14]
	▪ Perceived support from HCPs [5, 45]
	**Information overload and sensitisation** [15]
	**Positive psychological support**
	▪ Encouragement [14, 37, 38, 46, 58, 59, 61, 65]
	▪ Empowerment [42, 64]
	▪ Motivation [39, 40, 43, 49–52, 58–60, 64, 66]
	▪ Knowledge gained improved self-efficacy and confidence [40, 45, 52]
Outcomes	**Improved overall health-seeking behaviour (O+)**
	▪ Improved MCH care [14, 42, 51, 59–61, 64–66]
	▪ Improved use of ANC and PNC [5, 37, 38, 40, 43, 45, 46, 50, 52, 57, 58]
	▪ Improved SBA, facility birth and emergency obstetric care [5, 14, 37–39, 45, 46, 50, 52]
	▪ Increased use of iron tablets and immunization [56, 58]
	**Decreased visits to health facilities based on perceived desensitization (O-)** [15]

For more details on thematic analysis (Supplementary File 2, Tables 3 and 4).

### Implementation of mHealth by CHWs and HCPs

**Fig. 3:**
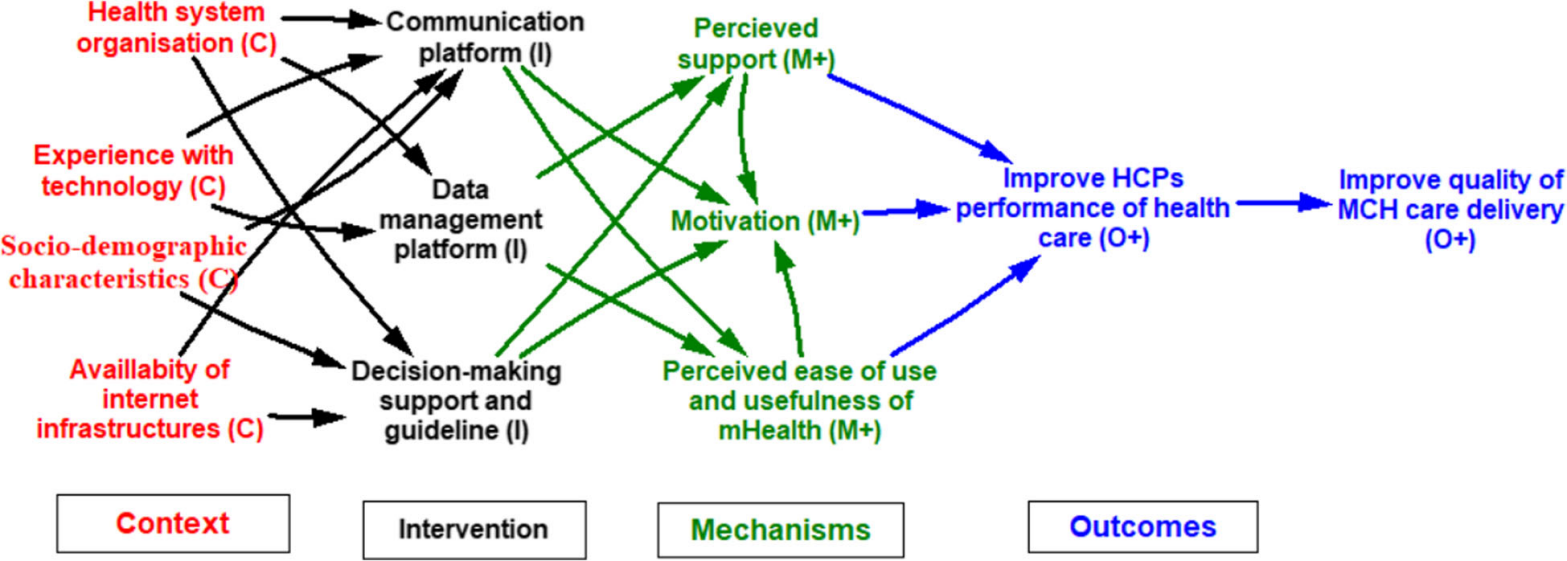
Configuration model on how and why mHealth works for HCPs

Table 3 presents the themes used to map the HCPs ICAMO (Fig. 3), which shows an explanatory model of how and why HCPs implement mHealth interventions (or not).

The first aspect of mHealth interventions is that it offers ‘communication platform’ (I) [37–42, 45, 54]. This is influenced by ‘health system organization’(C) [5, 37, 45], their ‘experience with technology’(C) [37, 41], HCPs’ socio-demographic characteristics (C) [54] and availability of internet infrastructure (C) [50]. Having a functional ‘communication platform’ motivates (M+) [38] HCPs to ‘improve their performance of health care’ (O+) [38, 50], which increases the quality of MCH care (O+) [38]. Also, the communication platform improves ‘perceived ease of use and usefulness of mHealth’(M+) [46, 47], which also improves their performance of health care (O+) [47].

The second relevant aspect of mHealth interventions relates to their ability to offer a ‘data management platform’ (I) [14, 45, 46, 48, 51]. The importance of data management platform is influenced by health system organization (C) [14, 45] and experience with technology (C) [41]. Having a functional data management platform improves perceived support of HCPs (M+) [51], resulting in improved HCPs’ performance of health care (O+) [51]. Also, the data management platform facilitates the perceived ease of use and usefulness of mHealth (M+) [46, 47], leading to improved HCPs’ performance of health care (O+) [46, 47].

Another important aspect of mHealth interventions for /HCPs is that these offer an environment of ‘decision-making support and guidelines’ (I) [50–53]. Decision-making support and guidelines are influenced by ‘health system organization’(C) [50, 52] socio-demographic characteristics (C), and ‘availability of internet infrastructure’(C) [50]. Having decision-making support systems and guidelines motivate HCPs (M+) [50, 53], thus improving performance of health care (O+) [50, 53] and quality of MCH care (O+) [53]. Finally, decision-making support and guidelines improve perceived support (M+) [51] and result in improved performance [51] and hence the quality of MCH care (O+).

### Uptake and outcomes of mHealth for pregnant women and mothers

**Fig. 4:**
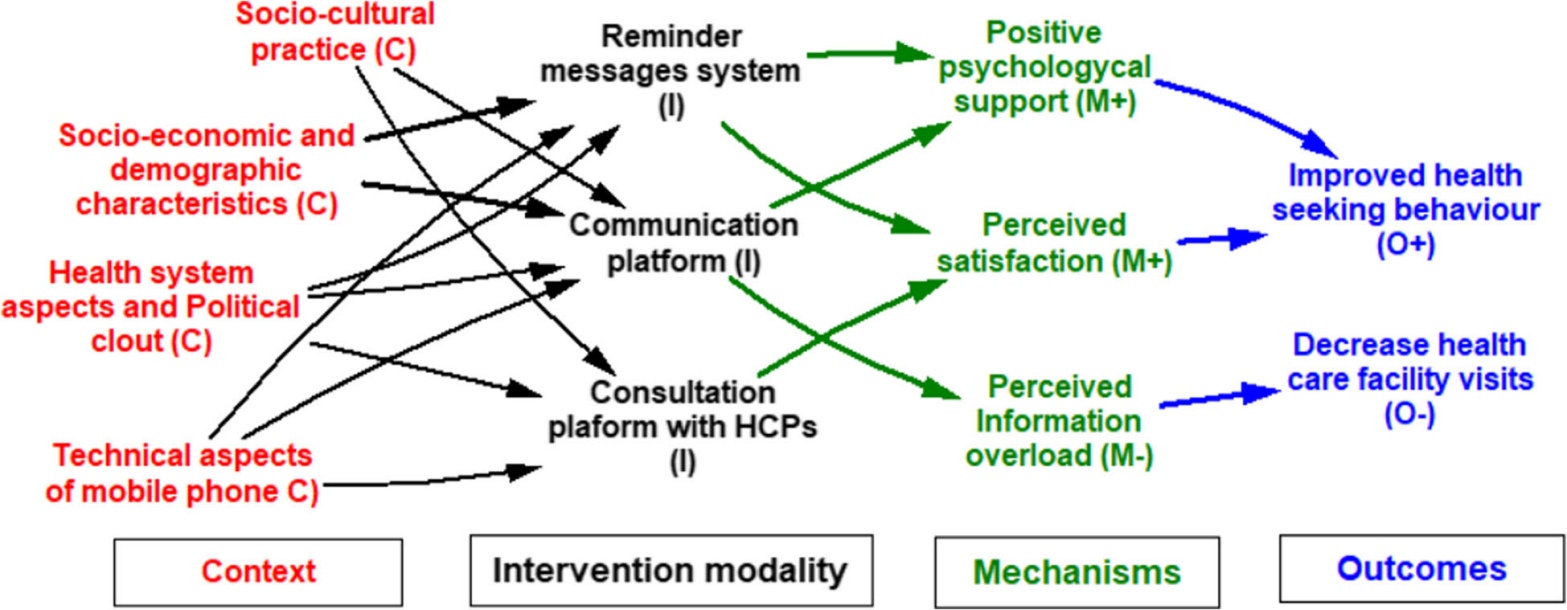
Configuration model of how and why mHealth works for pregnant women and mothers

Table 4 presents relevant themes used to develop the ICAMO model for pregnant women and mothers while Fig. 4 presents a model illustrating how and why various aspects of mHealth interventions work for pregnant women and mothers.

The first important aspect of the uptake of mHealth interventions by pregnant women and mothers is the reminder messages system (I) [37, 50, 52, 55–60]. This aspect is influenced by socioeconomic and demographics characteristics (C) [37, 50, 52, 55–57, 59]; health system and political clout [37], and technical aspects of mobile phone services (C) [50, 55, 56, 58]. Having reminder message systems improve ‘positive psychological support’(M+) [37, 50, 52, 58–60] and ‘perceived satisfaction of care’ (M+) [56, 60], resulting in improved health-seeking behavior (O+) [37, 50, 52].

mHealth interventions also provide a communication platform (I) for pregnant women and mothers [5, 14, 15, 38, 42, 43, 45, 46, 49, 50, 59, 61–65], which is influenced by socio-cultural practices and norms (C) [14, 64], socio-economic and demographics characteristics (C) [14, 15, 42, 46, 50, 59, 62, 64, 65], health system and political clout (C) [5, 38, 43, 45, 49, 61, 63] and technical aspects of mobile phone services (C) [15, 61, 63]. The communication platform improves ‘positive psychological support’(M+) [14, 38, 42, 43, 45, 46, 50, 59, 61, 64, 65], thereby improving health-seeking behavior (O+) [14, 42, 45, 59, 61]. For instance, when users are educated about MCH care, their capabilities to make healthy choices are enhanced, which motivates them to seek medical care on time [64]. Nevertheless, perceived information overload (M-) can result in decreased visits to health facilities based on desensitization, as pregnant women and mothers who have access to more information online and on their mobile phone may become complacent with using health facilities (O-) [15].

mHealth interventions also offer a ‘consultation platform with HCPs (I) [39, 48, 51], which is influenced by socio-cultural practices (C) [51], health system and political clout [39, 48], and technical aspects of mobile phone services (C) [39]. The consultation platform improves perceived satisfaction of care (M+) [14, 48] and health-seeking behavior (O+) [39, 51].

**Fig. 5:**
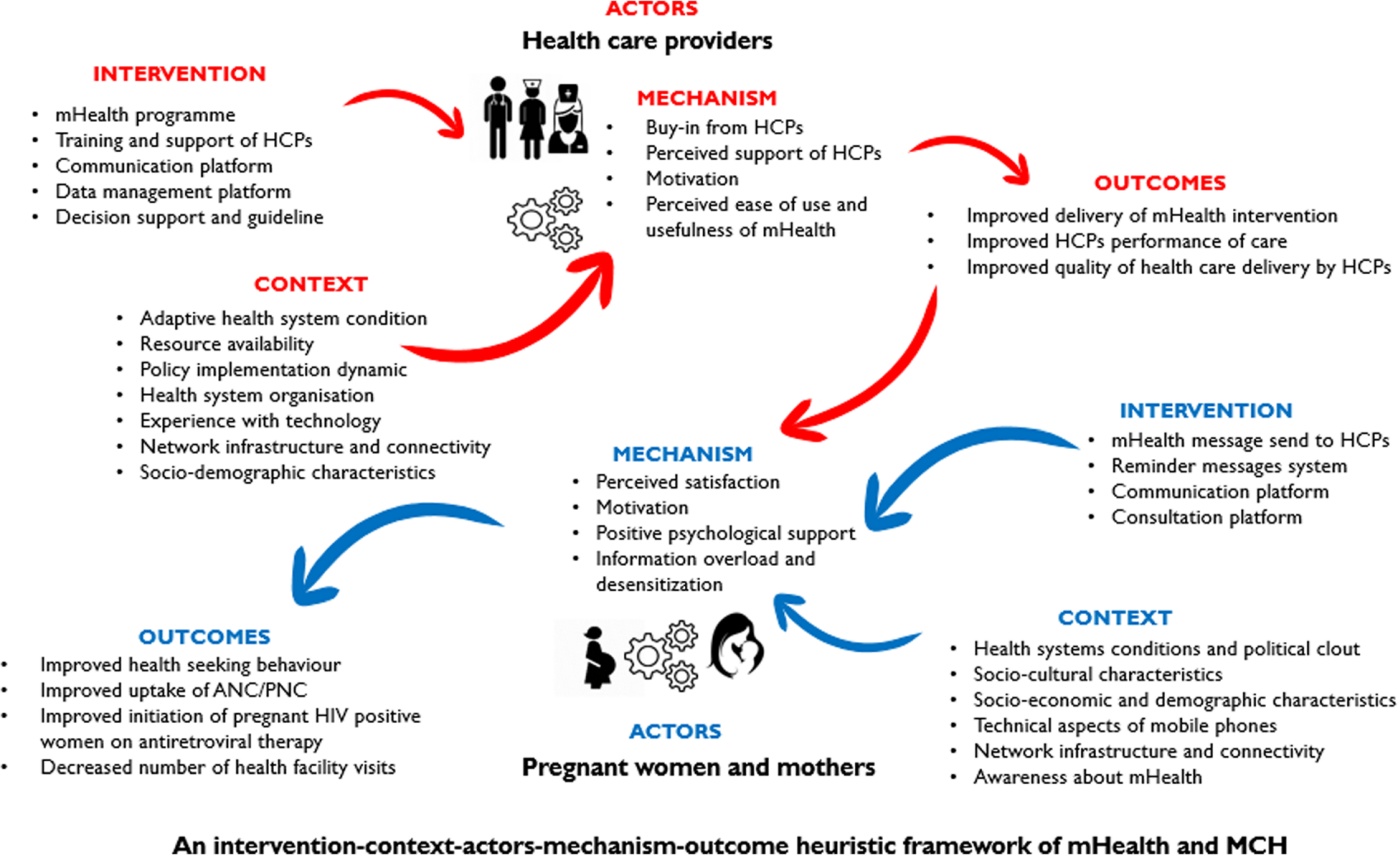
Program theory of mHealth programs and MCH

We combined the tentative programme theory (Fig. 1), the HCPs model (Fig. 3), and the pregnant women and mothers model (Fig. 4) to create a mHealth program theory (Fig. 5), which portrays how adoption of mHealth programs by HCPs and pregnant women and mothers’ influences performance and quality of health care among HCPs and health-seeking behaviors among pregnant women and mothers. We identified that performance and quality of service by HCPs (O+) were influenced by four different mechanisms: (1) Buy-in from HCPs (M+), explaining that HCPs’ engagement with mHealth impacted their performance. (2) Perceived support of HCPs, which shows how the perceived support of HCPs such as quality of training, resources, and administrative support help HCPs to perform their task and improve the relationship between HCPs and community. (3) Motivation (M+), reflecting how mHealth encourages HCPs to be more active in their task and how knowledge acquisition and skills improve self-efficacy and confidence. (4) Ease of use and usefulness of mHealth (M+), which shows how the quality of training is received, resource availability, administrative support, knowledge and skills gained helped to improve their tasks such as data collection and data management.

In addition, four mechanisms explained how the mHealth program influences health-seeking behavior among pregnant women and mothers including: (1) Perceived satisfaction (M+), explaining how perceived privacy, confidentiality and support from HCPs can influence health-seeking behaviors. (2) Motivation (M+), reflection of how information and education received through mHealth act as a stimulus for health-seeking behaviors. (3) Positive psychological support (M+), reflecting how knowledge gained improved self-efficacy, confidence, empowered and motivated pregnant women and mothers can impact on health-seeking behaviors. (4) Information overload and sensitization (M-), reflecting how accessing MCH information has positive or negative effect as pregnant women and mothers may become complacent to using health facilities once they can access this through their mobile phones [15].

The model shows that improved performance and quality of health care by HCPs (O+) have an impact on the mechanisms activated by pregnant women and mothers to produce the outcomes.

## Discussion

The present realist synthesis analyzed 32 articles describing eight intervention modalities used to implement mHealth programs for HCPs, pregnant women and mothers in LMICs, namely mHealth programs, training and support of HCPs, communication platform, data management platform, decision support and guidelines (for HCPs), mHealth messages sent to HCPs, reminder messages system, communication platform, consultation platform (for pregnant women and mothers). Our findings are supported by many other studies [44, 46, 48, 59].

This review unearthed theoretical models explicating how the adoption of mHealth program by HCPs, pregnant women and mothers can improve MCH. Our findings on the two models ICAMO resume that improved delivery of mHealth program, performance of care, and quality of health care by HCPs (O+) will influence the perceived satisfaction, motivation and psychological support (M+) of pregnant women and mothers (A) that will in turn influence on their overall health-seeking behaviors (O+). Our finding that context influences adoption of mHealth by HCPs concurs with those of Abejirinde et al. [48], which showed that mHealth empowered HCPs to adopt and use mHealth in contexts where it aligns to with needs, workload, training and skills. Perceived usefulness and ease of use of mHealth encouraged HCPs with skills and confidence, perceived usefulness related to design and technical concerns, cost, time, privacy, ease of use, security issues, risk-benefit assessment, experience with technology and contact with colleagues and patients [67, 68].

Our model is supported by Azhar and Dhillon [68] that identified behavioral intent, self-efficacy, social influence, attitude and perceived privacy threat as factors that influenced successful use of mHealth applications for self-care [68]. Moreover, our model is supported by a systematic review by Aker et al. that found that users’ perceived platform quality, perceived satisfaction of care, perceived quality interaction and outcomes, influence users’ uptake of mHealth for health care [69]. Our findings are supported by a realist-informed document review that identified empowerment, perceived quality of MCH care, encouragement, motivation, and knowledge acquisition as the main mechanisms driving the implementation and uptake of MCH care through the MomConnect program [70].

### How does our model compare to relevant existing frameworks?

The Fogg Behavior Model (FBM) [71] is a psychological model which proposes that for a targeted behavior to occur, presence of the following is needed in tandem for a target behavior: Ample motivation, ability and an active trigger. The Fit between Individual, Task and Technology (FITT) framework explains the degree to which technology’s functionality matches task requirements and individuals abilities to use technology to perform tasks [72]. The Technology Acceptance Model (TAM) seeks to explain users’ adoption or rejection of information technology by focusing on two theoretical constructs: perceived ease of use and usefulness [73]. According to TAM, if potential users believe an application is useful, they may at the same time believe that the system is easy or not easy to use, which makes the performance of benefit of usage outweigh the effort of using the application [73].

We found that FBM, FITT and TAM identified constructs that could be considered by realists as mechanisms to explain how mHealth interventions work. For instance, the FBM model revealed user motivation as central for how mHealth interventions work, whereas the FITT model highlights the perceived ease of use as central mechanism of how mHealth interventions work. The TAM model on the other hand, reveals perceived ease of use and usefulness as central ingredients to intervention uptake. While using theoretical frameworks in mHealth evaluation has been found beneficial to inform best practices [74], these models are limited in their explanatory power, because they largely ignore contextual elements in triggering identified mechanisms. Our ICAMO models thus not only identify further mechanisms and relevant contextual elements but also illustrate how contextual factors could impact on intervention modalities to activate mechanisms that produce outcomes. In this way, our models do not only provide evidence of how and why mHealth interventions work or not, but also context-linked explanatory theories to inform implementation and rollout of mHealth interventions to ensure conducive health systems and programmatic conditions that increase the chances of uptake among users.

### Strengths and limitations

Understanding the influence of mHealth by focusing on mechanisms and contextual factors through which outcomes are generated, is relevant because more information can be obtained about why mHealth interventions work or not and what triggers observed outcomes. Lack of information on how mHealth interventions work may encumber understanding of challenges and justifications for the implementation of successful mHealth programs, as well as its limitations.

A limitation of this review is that only six databases were searched and that search terms were restricted to LMICs, which could potentially bias the findings. The review also relied only on open access articles or those accessible through the electronic database and search engines published in English, which could have resulted in missing important studies on mHealth interventions for MCH care. Most articles did not conceptualize notions of context and mechanisms as understood in a realist philosophical sense. Thus, strict identification of these concepts needed further interpretation (abduction thinking) by the authors. Published studies on MCH-allied mHealth programs are growing, but have been inadequate in evaluating context and mechanisms by which outcomes are generated. More research is needed to evaluate mHealth using realist methods by comparing higher and LMICs.

## Conclusion

This review unearthed theoretical models explicating utilization of mHealth by HCPs and pregnant women and mothers. The models developed in the study provide detailed understanding of the uptake of mHealth interventions and how they enhance MCH care in LMICs. Our findings suggest that mHealth programs can shift the pattern of health care utilization and can be applied by policymakers to inform implementation strategies for mHealth programs in LMICs. By making explicit ICAMO configurations that are associated with success and failure of mHealth programs, policymakers can be informed on critical aspects that can inform scale-up of mHealth interventions. ICAMO models can yield important insights into potential policy changes that need to be enacted for mHealth interventions to be successful at scale. These models provide a foundation for the ‘white box’ of theory-driven evaluation of mHealth interventions and hence improve implementation where required.

## Supplementary Information

**Additional file 1.** Research Evidence Extraction/appraisal tool. This tool assisted in assessing the quality of studies included in the review.

**Additional file 2.** Part a) HCPs data charting, and Part b) pregnant women and mothers. This file provides two tables of information on ICAMO data extraction.

## Data Availability

The dataset(s) supporting the conclusions of this article is (are) included within the article (and its additional file(s).

## Abbreviations

ANC/PNC: Antenatal and postnatal care
CHWs: Community Health Workers
CMOc: Context-mechanism-outcome configuration
FITT: Fit between individual, task and technology
HCPs: Healthcare providers
LMICs: Lower- and Middle-Income Countries
MCH: Mother and Child Health
mHealth: mobile Health
NS: Narrative synthesis
ICAMO: Intervention-context-actors-mechanism-outcomes
TAM: Technology acceptance model
RCT: Randomized controlled trials
SDGs: Sustainable development goals
SMS: Short messaging services
