# Supplementary material for: Explaining the impact of mHealth on maternal and child health care in low- and middle-income countries: a realist synthesis
Source: BMC Pregnancy Childbirth. 2021 Mar 9;21:196. doi: 10.1186/s12884-021-03684-x (PMC7941738; doi:10.1186/s12884-021-03684-x)
Supplement: Supplementary file 1 — Additional file 1. Research Evidence Extraction/appraisal tool. This tool assisted in assessing the quality of studies included in the review. [file 12884_2021_3684_MOESM1_ESM.docx]

| **Analyse Phase** | | | | |  | | | |
| --- | --- | --- | --- | --- | --- | --- | --- | --- |
| Article Title | | | | | | | | |
| Authors |  | | | | | | | |
| Journal |  | | | | | | | |
| Setting |  | | | | | | | |
| Sample Size |  | | | | | | | |
| Objective of study clearly stated | | | | |  | | | |
| Study methodology or methodologies used | | | | |  | | | |
| Inclusion of sufficient data to assess validity of conclusions | | | | |  | | | |
| Data source | | | | |  | | | |
| Size of achieved sample and the population from which the sample is drawn | | | | |  | | | |
| Methods of measurement (data collection) | | | | |  | | | |
| Effect on behaviour | | | | |  | | | |
| Study type | - Meta-analysis | | - Quasi-experimental | | - Non-experimental | | - Qualitative | - Meta-synthesis |
| Does this study apply to the population targeted the review question? | | | | | | | - Yes | - No |
| Strength of the study design | | | | | | | | |
| Is the sample size adequate and appropriate? | | | | | | | - Yes | - No |
| Are the study participants randomised? | | | | | | | - Yes | - No |
| Is there an intervention? | | | | | | | - Yes | - No |
| Is there a control group? | | | | | | | - Yes | - No |
| If there is more than one group, are the groups equally treated except for the intervention? | | | | | | | - Yes | - No |
| Is there an adequate description of the data collection methods? | | | | | | | - Yes | - No |
| Study Results | | | | | | | | |
| Are the results clearly presented? | | | | | | | - Yes | - No |
| Is the interpretation/analysis provided | | | | | | | - Yes | - No |
| Study Conclusions | | | | | | | | |
| Are the conclusions based on clearly presented results? | | | | | | | - Yes | - No |
| Are the study limitations identified and discussed? | | | | | | | - Yes | - No |
| Comment: | | | | | | | | |
| Will the results answer the review questions? | | | | | | | - Yes | - No |
| Evidence Rating | | | | | | | | |
| Strength of Evidence | | - Level I | | - Level II | | - Level III | - Level IV | - Level V |
| Quality of Evidence (Check one) | | | | | | - High (A)x | - Good | - Low (c) |

**Research Evidence Extraction/appraisal tool**
