# Supplementary material for: Explaining the impact of mHealth on maternal and child health care in low- and middle-income countries: a realist synthesis
Source: BMC Pregnancy Childbirth. 2021 Mar 9;21:196. doi: 10.1186/s12884-021-03684-x (PMC7941738; doi:10.1186/s12884-021-03684-x)
Supplement: Supplementary file 2 — Additional file 2. Part a) HCPs data charting, and Part b) pregnant women and mothers. This file provides two tables of information on ICAMO data extraction. [file 12884_2021_3684_MOESM2_ESM.docx]

**Supplementary File 2**

**Part a. HCPs data charting**

| **Author**  **Year/country** | **Aim of the study and intervention** | **Intervention modalities** | **Context** | **Actors** | **Mechanisms** | **Outcomes** |
| --- | --- | --- | --- | --- | --- | --- |
| 1.Musabyimana et al. (2018)(Musabyimana et al. 2018)  Rwanda | - To improve access to ANC/PNC, institutional delivery and emergency obstetric care in Rwanda. | - Rapid appointment reminder SMS.  -Training of community health worker  -communication platform | -Insufficient training,  - lack of equipment  -Technical aspects of phone service | -Key stakeholders.  -HCPs  -CHWs | - Lack of CHW motivation.  - lack of encouragement | -mHealth was adopted by both CHWs and parents  - Respondents believed that RapidSMS contributed to reducing maternal and child mortality rates (improve MCH). |
| 2.Ruton et al., 2018(Ruton et al. 2018)/Rwanda | -A longitudinal study to retrospectively evaluate the impact of Rapid SMS both with and without this additional support on the use of important MCH services | -Health education platform | -Continuous training, supervision and the provision of medical equipment. | -Community health workers. | - Perceived training of CHWs, equipment and supervision provide CHWs with knowledge to increase the quality of their services | - Increase the quality of collaboration between CHWs and community members |
| 3.Kaewkungwal et al2010 (Kaewkungwal J. et al. 2010)/ Thailand | -To assess the application of cell phone integrating into the healthcare system to improve ANC and expanded programme on immunization (EPI) services | -Update information regarding the ANC and child’s immunization status.  -Education Platform | -Training of HCP to understand and apply mobile phone as part of their routine works. | HCP who oversaw ANC/EPI activities | Not found | -Increased in access rate to PNC and contraception (performance) |
| 4.Ngabo et al 2012(Ngabo et al. 2012)Rwanda | -Describes the design and implementation of a mobile phone-based communication system aiming at monitoring pregnancy and reducing the three delays in communication | -Rapid appointment SMS  - Communication platform, between CHWs at community level, and the rest of the health system. | -CHW were trained and equipped with mobile phones  -District heath team capacity to manage and supervise the system was strengthened | -CHWs | - Phone distribution was perceived as a guarantee to boost engagement and  motivation of CHWs | -CHWs reported being more pro-active in finding new pregnant women and follow up. |
| 5.Tobe et al. 2018(Tobe et al. 2018)/Bangladesh | The intervention was implemented to improve maternal and child health care for targeting SDGs. | -Mobile short messaging and audio system  -Information and education  -Preservation of home-based health records  -Facilitation of continuum of perinatal care. | -Staffs use phone call instead of short messages (for those who cannot read).  Literacy | -(CHWs), skilled birth attendants (SBAs) staffs | -The information received encouraged participants to be more active in doing their task. | -Improved CHWs performance |
| 6.Mwendwa 2016 (Mwendwa 2016)  /Rwanda | To examines the challenges and opportunities faced by CHWs who use a mHealth tool. | -mHealth to support CHWs tasks  -CHWs register new pregnancies into  the system and report all danger signs. | -CHWs mobile phones distribution  -Inadequate training and supervision  - CHW skills, resources, and administrative support.  - level of education | -CHWS | -Perceived ease and usefulness of use, quality of training, CHW skills, resources, and administrative support  impact on respondents’ task. | -Improved CHWs skills  -Helped CHWs to overcome the different barriers faced in the field |
| 7.Bigna et al.(2014)/(Bigna et al. 2014)Cameroun | To assess whether reminders sent to carers by text message, mobile phone call and mobile phone call increase attendance at medical appointments | -Mobile-phone-based reminders  -Text messages and call phones  -Communication platform | -Staff working time  -Level of education | -HCPs | - | -CHWS increased their performance by increasing the rate of health care attendance |
| 8.Ilozumba et al. (2018)/(Ilozumba et al. 2018)India | -To understand how the mHealth intervention was implemented and to gain insight into contextual factors that potentially influenced the observed intervention outcomes | - Apps (Mobile for Mothers (MfM)).  -Health education  -Data collection | -Training of CHWs  - The mobile pre-loaded with phone credit and provided free of charge to CHWs.  -CHWs support | CHWs who delivered the intervention. | -Perceived support helped CHWs to acquire the knowledge and improve their self-efficacy and skills in doing their duties | Improved utilisation of MCH services |
| 9.Hackett et al. (2018)/(Hackett K. et al. 2018) Tanzania | To evaluate a smartphone-based application designed to assist community health workers with data collection, education delivery, gestational danger sign identification, and referrals. | -Apps  - Data security and privacy | -Support | -CHWs | - mHealth helped CHWSs to gain Skill and knowledge such as social and technical capacity to work in confidence  Positive impacts on perceptions of personal privacy and confidentiality |  |
| 10.Asiki etal.2018(Asiki et al. 2018)/Uganda | To contribute information to future trials for improvement of child survival in the critical period surrounding birth and by pregnant women | Apps (doForm and paper forms)  -Data management: registration and tracking of pregnant women | -Training of VHWs on the use of smartphones for pregnancy registration.  -Ability of the VHWs to use the smartphone apps. | -CHWs | -Perceived usefulness of mHealth helped CHWs to improve their knowledge and skill in data collection and management | -Health workers were enthusiastic with the use of the phones and performed well |
| 11.McNabb et al., 2015(McNabb et al. 2015)/ Nigeria | -To assess the impact of the quality of services offered as a result of the intervention as well as the client satisfaction with services offered using the mobile phone application | -APPs (CommCare mobile platform)  -Decision support and data collection platform | -Shortage of HCPs  - Low skill level of CHEWs and lack of adequate supervision, and technical support adversely affects the quality of health services | -CHWs | Improved knowledge among CHWs | - m4Change intervention was in fact associated with higher quality of ANC scores  -A significant improvement in overall client satisfaction. |
| 12.Kaphle et al. 2015(Kaphle et al. 2015)/India | To develop a framework to assess whether mHealth platforms affect the quality and experience of maternal and newborn care provided by CHWs | - APPs (CommCar)  -Health education and promotion. | - Level of mHealth technology adoption  - Attitude and training  -Socio- demographics characteristcs | -CHWs | -Level of technology adoption was important for both quality and experience of care.  -The message provided through images, audio, and vide encouraged CHWs to improve their behavioural communication | - Highr users have a Significantly higher scores for quality and experience of care compared to low users.  - Age (-)  -Literacy level (-)  -Education (-) |
| 13.Balakrishnan et al 2016/ (Balakrishnan et al. 2016) India | To evaluate the overall feasibility and efficacy of using mHealth technology for delivering the continuum of MCH services | -Apps (health information delivery) | -Front Line Workers (FLW) training on mobile application | -Accredited Social Health Activist (ASHA) and the latter (is the Anganwadi) | -Perceived knowledge acquisition among ASHA | -Improves service delivery and strengthen the Health System. |
| 14.Atnafu et al. 2017(Atnafu, Otto, and Herbst 2017)/Ethiopia | To determine whether the mobile phone SMS based data exchange application influencing health extension workers (HEWs) can improve MCH services | -Frontline SMS based application.  - Decision support | -Mobile phone distributed to HEWs | -HEWs | -The interaction of HEWs helped to improve access to ANC, Delivery Services, and PNC (Sel-efficacy) | - Increases in referrals from health post to health center by HEWs,  -Increased in ANC/PNC service delivery |
| 15.Hackett et al 2018(Hackett, Kazemi, and Sellen 2018)/Tanzania | To explores user and beneficiary perceptions of data security and privacy within the context of a smartphone intervention to improve women's uptake of MCH services. | -APPS  -Guiding CHWs through electronic protocols and directing them to specific health messages and counselling topics | -Training on the smartphone application along with the photo flipbooks. | -CHWs | - Perceived support enhancing relationship between female clients and CHWs, so long as clients believe their health information and reproductive health “secrets” will be stored securely. | -Increased in data security, privacy, and confidentiality.  - Closeness between clients and CHWs has increased as women feels comfortable to tell the secrets that she cannot tell anyone else. |
| 16.Martinez et al.,2018(Martinez et al. 2018)Guatemala | To characterize, baseline rates of complication detection and facility-level referral by TBAs in rural Guatemala, and to evaluate the impact of the mHealth system on these rates. | -Apps (Maya Health Alliance (MHA  -Decision support and guideline | -Guatemalan primary health care organization  -A study staff member bilingual in Kaqchikel and Spanish subsequently visited patients and the biological father, when available | -HCPs | The APPs Motivated TBAs to improve their task | -Increase in emergency referrals rate among HCPs who has access to the technology,  -Improving the early detection of complications in community-based care. |
| 17.Ayiasi et al 2015/ (Ayiasi et al. 2015)Uganda | To investigated perceived maternal and newborn benefits of home visits made by village health team (VHTs), combined with mobile phone consultations with professional health workers for advice. | -Communication plat form  -Mobile phone consultations with professional health workers for advice. | -Home visits made by VHTs  -Resistance from part of the professional health workforce to collaborate with VHTs.  -Unavailability of professional health workers. | -CHWs also called VHTs | -Knowledge gained gave VHT confidence to carry on their duties and facilitated communication between them and professional health workers in MCH. | -Increase in knowledge among VHTs to do their duties |
| 18.Shiferaw et al. 2016/(Shiferaw et al. 2016) Ethiopia | To determine whether an mHealth intervention and training of health providers on client centered care, can improve maternity service utilization | - Decision support  -Report module | -Training of HCWs  -Owned a mobile phone. | -Health workers | - The use of mobile phone encourages HWs in performing their duties, by calling pregnant women to come on their appointment. | - Improved MCH services delivery |
| 19.Modi et al. (2015)/ (Modi et al. 2015) India | To describes the process of development and  formative evaluation of a complex intervention (called ImTeCHO) based on mobile phone technology (mHealth) | -SMS text and video.  -Health information delivery | -ASHAs support and mobilization of pregnant women for ANC,  -Escorting pregnant women to the hospital for delivery, | -Accredited Social Health Activist  (ASHA). | - Adequate support of ASHA and supervision motivate the frontline health workers to performance well their duties. | - Increased in assistance with early breastfeeding, and identification of sick new-borns. |
| 20.Abejirinde et al, 2018(Abejirinde et al. 2018)/Ghana | To explores the experiences of women exposed to the B4M device, in order to find out their experience of the use of Bliss4Midwives during their routine ANC consultations, and what influence did Bliss4 Midwives have | -mHealth device (Test result turnaround) Bliss4Midwives (B4M)  - Diagnostic and treatment  -Data collection platform | -HCPs training | -Health workers | - Perceived skills improvement and knowledge of health workers, facilitated trust in diagnostic recommendations and was therefore believed to motivate referral compliance. | - Increase the use of health services and referral recommendation.  - The interpersonal process of care and component of quality ANC is improved. |

**Part b. Pregnant women and mothers**

| **Author**  **Year/country** | **Aim of the study and intervention** | **Intervention modalities** | **Context** | **Actors** | **Mechanisms** | **Outcomes** |
| --- | --- | --- | --- | --- | --- | --- |
| 1.Ruton et al., 2018(Ruton et al. 2018)/Rwanda | A longitudinal study to retrospectively evaluate the impact of Rapid SMS both with and without this additional support on the use of important MCH services | -SMS text message  -Information on ANC, PNC and facility delivery | -Health system capacity  -Support including training,  supervision and equipment provision | -Pregnant women | Perceived support motivated women to improve their MCH services uptake. | -ANC/PNC increased  -Increase in the trend of facility deliveries in supported Districts compared to non-supported districts.  -Increased use the uptake of MCH services |
| 2.Kaewkungwal et al.,2010(Kaewkungwal J. et al. 2010)/ Thailand | To assess the application of cell phone integrating into the healthcare system to improve ANC and expanded programme on immunization (EPI) services | -Reminder of mother and child on ANC/EPI visit due dates,  -Educational text message | -Updated immunisation date on cell phone  -Ethnicity, age, education and parity of pregnant (-) | -Pregnant women and mothers in the three cohorts no Thai including Karen, Myanmar and Mon. | -The educational text messages encouraged women to use health facility for delivery and bring their children to clinic after delivery. | -Increase in deliveries by SBA  -Increased in more than four ANC visits. |
| 3.Kazi et al 2017(Kazi et al. 2017)/ Kenya | -To assess the feasibility of SMS text messaging–based mHealth intervention intended to connect expectant mothers and child caregivers with their HCPs | - SMS text messaging (short messaging service, SMS) based communication on the rise  -Health information and education | -Mobile phone access  - Lower levels of literacy  -Reliable network connections.  - Preferred SMS text. | -Pregnant women  -caregivers attending the routine immunizations clinics for children | -Perceived shared phone arrangement helped to reach to the population with no phone thus women were satisfied to benefit from mHealth programme | -Increase in health services utilisation |
| 4.Nie et al. 2016(Nie et al. 2016)/Timor-Leste | -To explore the association between access to mobile phones and the utilization of health services related to pregnancy and childbirth | -SMS text reminder | - Socioeconomic status  -Mobile phone ownership | -Women aged 15–49 years old with a child up to  24 months | -No identified | -Improved utilisation of health care service among women exposed to mobile phone compared to those unexposed |
| 5.Ngabo et al 2012(Ngabo et al. 2012) Rwanda | -Describes the design and implementation of a mobile phone-based communication system aiming at monitoring pregnancy and reducing the three delays in communication | -Rapid appointment SMS  - Communication between a CHW following mother-infant pairs in their community.  -Communication platform | -Availability CHWs in charge of MCH services.  -Sensitize community about the needs to attend ANC  -Provide support and referral to the health in case of complication | -Pregnant women. | -CHWs communication encouraged women to attend ANC and have information on ambulance alert service | -Improved access to ANC/PNC, institutional delivery, and emergency  obstetric care. |
| 6.Tobe et al. 2018 (Tobe et al. 2018)/Bangladesh | The intervention was implemented to improve maternal and child health care for targeting SDGs | -Mobile short messaging and audio system  -Mother and Child Handbook (MCH)  -Information and education  -knowledge  dissemination, promotion of communication | - Shared cell phone  -Literacy | Pregnant women | -Women are empowered by the network to use MCH services at the community’s level | -Health seeking behaviour are improved among pregnant women |
| 8.Ilozumba et al. (2018)/(Ilozumba et al. 2018)India | To understand how the mHealth intervention was implemented and to gain insight into contextual factors that potentially influenced the observed intervention outcomes | -Apps (Mobile for Mothers (MfM)).  -The application on the mobile phone included a multimodal components text, image and audio.  -Health education | - Training of CHWs  - The mobile was pre-loaded with phone credit and provided free of charge to CHWs.  -CHWs support | -Pregnant and lactating women | -Perceived support by CHWs’ helped women to acquire the knowledge.  Knowledge gained improve their self-efficacy in seeking health during and after pregnancy. | Improvement in use of ANC and clinic deliveries in the intervention group compared to the control groups |
| 9.Hackett et al. (2018)/(Hackett K. et al. 2018)  Tanzania | To evaluate a smartphone-based application designed to assist community health workers with data collection, education delivery, gestational danger sign identification, and referrals. | -Apps  -Health counselling topics and messages based on the woman's gestational age, and her answers to various diagnostic questions.  -Education delivery | - Acceptability of new technologies  -Beliefs and gender dynamics  -Socio-economic factors | -Pregnant women | -Perceived privacy and confidentiality encouraged or help women to access facility-based clinical services. | -mHealth improve women's uptake of MCHs  - Significant higher rate of facility delivery. |
| 10.Asiki etal.2018(Asiki et al. 2018)/Uganda | - To contribute information to future trials for improvement of child survival in the critical period surrounding birth and by pregnant women | -Apps (doForm and paper forms)  - Health education  -MAMA message on antenatal care, safe delivery, nutrition and motivate mothers to get the right care at the right time. | - Mobile phone ownership  -Socio-economic factors. | -Women | - Phone ownership reinforce village health workers (VHWs) communication with pregnant women and this motivated them to change their health seeking behaviour | -Improvement in ANC/PNC, facility delivery |
| 11.Guerra-Reyes et al 2016(Guerra-Reyes et al. 2016)/India | -To assess low-income mothers’ perceptions of their postpartum information needs | -APPs (Text4baby)  -Information delivery  - Messaging about ANC, PNC, facilities deliveries and Breastfeeding | -Constraints or inadequate Internet infrastructure  -Lack of awareness of the existence of postpartum apps | -Low income women and mothers residing in Monroe County with at least one biological child aged 48 months or younger | -Online sources of knowledge  and perceived reassurance, helping to normalize a stressful transition  experience. | - Low-income women were more likely to use interpersonal sources of information and less likely to use online sources of health information |
| 12.McNabb et al., 2015(McNabb et al. 2015)/ Nigeria | -To assess the impact of the quality of services offered as a result of the intervention as well as the client satisfaction with services offered using the mobile phone application | -APPs (COMM Care mobile platform)  -Health information on prevention against malaria, postpartum contraception, HIV, and newborn care. | - Baseline: higher client satisfaction relating to usefulness of information received, respectful treatment  by provider, and client’s intention to return to the facility | -Women | - Audio-recorded health counselling messages motivated women to change their health behaviours. | - clients who said they were very satisfied  with the ANC services, increased from 75% at baseline to 83% at endline |
| 13.Balakrishnan et al 2016/ (Balakrishnan et al. 2016)India | To evaluate the overall feasibility and efficacy of using mHealth technology for delivering the continuum of MCH services | -Apps (health information delivery)  - Message on early registration of pregnancy and ANC/PNC and early initiation of breastfeeding | - Provision of MCH care | -Women and mothers  -Women enrolled in PMTCT programs | -Increase in knowledge of women about maternal and child health. This encouraged or motivated a better health seeking behaviour. | - Increased in ANC/PNC in the implementation block compared to the control block |
| 14.Atnafu et al. 2017(Atnafu, Otto, and Herbst 2017)/Ethiopia | To determine whether the mobile-phone short message service (SMS) based data exchange application influencing health extension workers (HEWs) can improve MCH services | - Apps  -Reminder ANC/PNC, delivery, immunization and contraceptive | -Socio demographic characteristics | Reminder about the scheduled date of ANC visit, expected date of delivery, PNC, immunization schedule and vaccine and contraceptive stock status. | - Positive contribution of SMS based mobile phone intervention motivated women to improve their self-efficacy which resulted in increase in MCH services | - Increased in SBA and facility delivery.  - Increased ANC/PNC uptake |
| 15.Nyemba-Mudenda et chigona 2018/(Nyemba-Mudenda and Chigona 2018) Malawi | -To investigate the capability outcomes enabled through the capability inputs of access and use of a mobile phone system in maternal health. | -APPS (ICT)  -Mobile System for Safe Motherhood (MSSM) intervention.  -To improve access to health information and services for pregnant women  -Communication platform  . | -Education level  -Social or cultural practices, social structures, and norms.  - The education level of a woman influenced how the women understood and interpreted the messages and solutions received | Women | -Access to health information empowered women to improve their skills, confidence, and appreciation of communicating using mobile phones.  -Knowledge gained motivated women to seek medical care before a condition got critical. | -literacy enhanced communication, and ICT use for the mobile phone knowledge gained  -Improved Health capabilities (make health choices),  -Improved the seeking of health care services in good time, |
| 16.Hackett et al 2018(Hackett, Kazemi, and Sellen 2018)/Tanzania | To explores user and beneficiary perceptions of data security and privacy within the context of a smartphone intervention to improve women's uptake of MCH services | -APPS  - Client registration, follow-up, recognition of PNC and obstetric danger signs, referral to health facilities,  -Data management, and service delivery reporting.  -Mobile phone consultation | -Community by in  -Cultural norm  -Acceptance of mHealth  -community gender dynamics.  -Women's belief | -Pregnant women | -The intervention motivated women by improving their understanding and believes that their health information and reproductive health information will be stored securely. | - Closeness between pregnant women, mothers and CHWs  -Increased use of MCH services |
| 17.Ayiasi et al 2015/ (Ayiasi et al. 2015)Uganda | To investigated perceived maternal and newborn benefits of home visits made by VHTs, combined with mobile phone consultations with professional health workers for advice. | -Communication plat form  -Mobile phone consultations with professional health workers for advice. | -Home visits made by village health team (VHTs),  -Unavailability of professional health workers.  - Network and battery charging issues | -Women and mothers | -Interaction  between women, VHTs and professional health workers were perceived to motivate women and encouraged them to uptake facility delivery | -Increased in facility delivery |
| 18.Yamin et al. 2018(Yamin et al. 2018)/Afghanistan | To determine the perceptions of Afghan women regarding the use of mobile phones for supporting MCH, and the associated factors with those perceptions. | -Mobile phones  -Health-related information and medical reminders | - Owners cell phone  -Ability to read  -Preferred to receive reminders 2 days before the appointment date  - Cell phone ownership or routinely used a mobile phone and ability to read were about twice more likely to use a mobile phone for health support. | -Pregnant Women and mothers | - Perceived ease of use motivated women to use health care service | - Improved the uptake of MCH services |
| 19.Jennings et al., 2015(Jennings et al. 2015)/Nigeria | To examine if women with limited mobile phone access have differential odds of maternal knowledge and health service utilization as compared to female mobile phone users | -Mobile phone access  -Health information delivery | -Participate level of education  -Household wealthy quintile  - Lack of access to a mobile device | -Women | No mechanism found. | - Women without access to mobile phones were significantly less knowledgeable of the benefits of skilled maternal care and dangers signs |
| 20.Coleman et al, 2017(Coleman et al. 2017)/South Africa. | -To examine Whether the MAMA maternal mHealth intervention was an effective strategy for improving retention-in-ANC and increasing postnatal infant HIV testing and improving birth outcomes for HIV-positive women, and their infants, | -SMS (Health information delivery) -message on maternal health (e.g. healthy eating, reminders of ANC/PNC appointments, psychosocial support, delivery planning),  - HIV, PMTCT-related messaging (including PCR testing reminders and importance of adherence to ART. | -Socio-demographic | -HIV-positive women who attended their first ANC visit | - | - Increased the average number of ANC visits, improved the likelihood of attending, at least four ANC visits, and increased vaginal birth rates |
| 21.OBasola et al.(2017)/(Obasola and Mabawonku 2017)Nigeria | To provide a robust overall measure of how mothers use ICT to access MCH information, as well as its influence on their health practices | -ICT as a channel  for communicating MCH information  - Formats, such as voice, text, picture and video.  -Messaging on nutrition during pregnancy, family planning, disease prevention in pregnancy, Breast feeding, Medication in, Mental health and emotional changes in pregnancy. | -Poor mobile phone network.  - Unreliable power supply  - low incomes have cost issues | -Mother | - Information overload.  -The use of ICT by women in accessing MCH information  influence their health positively or negatively.  -Mothers may become complacent about the use of health facilities once they can access the required health information through ICT channels. | - Improve or reduce doctor-patient relationship  - women avoided visiting health facilities once they could access the needed MCH information through ICTs.  - High prevalence  of the use of mobile phones by mothers to access MCH information from health workers.  -Increase in the utilisation of health facilities. |
| 22.Shiferaw et al. 2016/(Shiferaw et al. 2016) Ethiopia | To determine whether an mHealth intervention and training of health providers on client centered care, can improve maternity service utilization | -Reminders of subsequent visits of ANC/PNC and delivery  -Health education message on dangers signs during pregnancy | -Literacy  -Owned a mobile phone. | -ANC and PNC women | -Women were motivated to have more frequent ANC visits.  - Women felt more valued by health workers and hence be responsive to the advice they received at health facilities 9 perceived satisfaction. | - Increased facility delivery and PNC service utilization regardless of age of women, residence  (urban/rural) and parity. |
| 23.Modi et al. (2015)/ (Modi et al. 2015) India | To describes the process of development and formative evaluation of a complex intervention (called ImTeCHO) based on mobile phone technology (mHealth) | -SMS text and video.  -Health information delivery | -ASHAs support and mobilization of pregnant women for ANC.  -Escorting pregnant women to the hospital for delivery, | -Pregnant women and mothers | - The intervention improved women confidence and they were motivated to deliver at health facilities through preparation of a birth plan, | - Higher uptake of ANC/ PNC and counselling. |
| 24. Abekah-Nkrumah 2014/(Abekah-Nkrumah, Guerriero, and Purohit 2014) Ghana | To use a demand side perspective to examine the effect of Information and Communication Technologies (ICTs) on the reproductive health services | ICTs (i.e. landline phone, listening to radio, watch television,  colour television in household, computer in household),  -Health information delivery | -mobile phone ownership  -Socio-economic characteristics of the mother, the household and the community,  The individual characteristics | Women | - ICTs influence  the demand for reproductive health services and women were encouraged to  gain access to health-related information. | -Increase in uptake of MCH service  Household wealth, woman’s age, years of education, regional and provincial differences, religion, ethnicity and access to health services influence the utilization of maternal health services. |
| 25.Skinner et al. 2018 (Skinner et al. 2018)/  South Africa | To assess the felt value of MomConnect, to describe the experiences of pregnant women and new mothers with MomConnect, and to obtain suggestions for its further improvement. | -SMS reminder  -Health educational text messaging service covering broad areas of MCH services were sent to pregnant mothers from the time they presented at the clinic.  -One to three messages a week, depending on the stage of the pregnancy | -Poor community  - High unemployment and poor housing.  - Appreciative and acceptance of the MomConnect programme. | -Pregnant women and new mothers | - The satisfaction with intervention motivated clients to plan around expected events and empowered them to decide when they needed to visit the clinic.  -Users feels empowered in their role as a mother, that enthusiastic about the messages lead them to use MCH services | -Women attached high value to the content of the messages and the medium in which they were delivered.  -They reported increased in the health services uptake. |
| 26.Bangal et al, 2017(Bangal et al. 2017)/India | The use of mobile phone in improvement of maternal health | -Mobile phone calls, as reminders about next visit and text messages (SMS) on important aspects of antenatal care at regular intervals.  -All women were advised for institutional delivery and to report for postnatal visits.  -Reminder and Education | -Mobile phone was owned by some close relative /family member staying with the pregnant woman.  -Mobile users were able to receive and reply to the phone call and text messages.  -Preference of languishes. | -Pregnant women | -Women are motivated and encouraged for institutional delivery and to report for postnatal visits. | - Increased ANC, consumption of iron tablets, tetanus toxoid immunization, institutional deliveries and PNC check-ups as compared to the control group. |
| 27.Feroz et al 2017(Feroz et al. 2017)/Pakistan | To explore the views, experiences, and perceptions of public HCP and pregnant and postnatal women from the peri-urban areas of Karachi regarding the utilization of mHealth intervention for enhancing ANC and PNC service uptake | -mHealth  -Communication platform  -Health information delivery. | - Government, local communities, telecommunication personnel, HCPs and mHealth and MCH experts (Gov. club).  -Luck of trust in technology and preference to meet doc face to face  -Literacy | -Pregnant women, women in the postnatal period and lady health workers (LHWs) | -Perceived communication ability encouraged women to use Health services  -mHealth improved their understanding and helped them in building their confidence. | -Increased in uptake of MCH services.  - mHealth provides guidance to patients through images, website links, inbound calls, outbound calls and messages. |
| 28.Barron et al., 2018(Barron et al. 2018)/ South Africa | MomConnect programme evaluation | -SMS reminder and educational, communication platform  -Messaging addressing behaviours associated with improved ANC, nutrition, HIV in pregnancy, exclusive breastfeeding and immunisation. | -Helpdesk allows women to ask individual questions (I)  -Feedback | -Pregnant women and mothers | -Users reported high levels of satisfaction with the messaging and this motivated them to use health care services.  -High value of system and the content of the messages motivated users to use MCH services. | -Increased in health services utilisation |
| 29.Abejirinde et al, 2018(Abejirinde et al. 2018)/Ghana | To explores the experiences of women exposed to the B4M device, in order to find out their experience of the use of Bliss4Midwives during their routine ANC consultations, and what influence did Bliss4 Midwives have | -mHealth device for consultation (Test result turnaround) Bliss4Midwives (B4M)  -Non-invasive device for measuring haemoglobin via infrared sensors mounted on a finger clip. | -Midwives and HPs trained on device  -Training on the principles of quality ANC and management of pregnancy complication | -Women | -Novelty effect of mHealth technological and appreciation for easier and additional diagnostic services gives women satisfaction. | -Pregnant women appreciated the device for detecting their health problems and saving time |
